# A Review of Research on the Association between Neuron–Astrocyte Signaling Processes and Depressive Symptoms

**DOI:** 10.3390/ijms24086985

**Published:** 2023-04-10

**Authors:** Jiao Yao, Cong Chen, Yi Guo, Yantao Yang, Xinya Liu, Shifeng Chu, Qidi Ai, Zhao Zhang, Meiyu Lin, Songwei Yang, Naihong Chen

**Affiliations:** 1 Hunan Engineering Technology Center of Standardization and Function of Chinese Herbal Decoction Pieces, College of Pharmacy, Hunan University of Chinese Medicine, Changsha 410208, China; 2 Key Laboratory of Modern Research of TCM, Education Department of Hunan Province, Changsha 410208, China; 3 School of Traditional Chinese Medicine, Hunan University of Chinese Medicine, Changsha 410208, China; 4 School of Acupuncture & Tuina and Rehabilitation, Hunan University of Chinese Medicine, Changsha 410208, China; 5 State Key Laboratory of Bioactive Substances and Functions of Natural Medicines, Institute of Materia Medica & Neuroscience Center, Chinese Academy of Medical Sciences and Peking Union Medical College, Beijing 100050, China

**Keywords:** depression, neurons, astrocytes, astrocyte–neuron interactions

## Abstract

Depression is a mental illness that has a serious negative impact on physical and mental health. The pathophysiology of depression is still unknown, and therapeutic medications have drawbacks, such as poor effectiveness, strong dependence, adverse drug withdrawal symptoms, and harmful side effects. Therefore, the primary purpose of contemporary research is to understand the exact pathophysiology of depression. The connection between astrocytes, neurons, and their interactions with depression has recently become the focus of great research interest. This review summarizes the pathological changes of neurons and astrocytes, and their interactions in depression, including the alterations of mid-spiny neurons and pyramidal neurons, the alterations of astrocyte-related biomarkers, and the alterations of gliotransmitters between astrocytes and neurons. In addition to providing the subjects of this research and suggestions for the pathogenesis and treatment techniques of depression, the intention of this article is to more clearly identify links between neuronal–astrocyte signaling processes and depressive symptoms.

## 1. Introduction

Depression is a worldwide psychological condition with a crisis rate of up to 20% [1]. One of the main signs of depression is a persistent gloomy mood, as well as an elevated risk of suicide, self-harm, and even violence [2]. Long-term denial about oneself is a common symptom of depression [2], which can strain family ties and result in significant financial, social, and familial burdens.

Numerous investigations into the causes of depression have been conducted, and numerous hypotheses have been proposed, including those regarding monoamine neurotransmitters and their receptors [3], hypothalamic–pituitary–adrenal axis dysfunction [4], neuroplasticity and neurotrophic factor [5], cellular molecular mechanism [6], inflammation and cytokines [7], and microbiota-gut-brain axis dysfunction [8], etc. Genetic, social-environmental, and other elements are considered as very important components of the pathophysiology of depression. In addition, depression is one of the most common complications in patients with type 1 diabetes (T1D) and type 2 diabetes (T2D). The risk of clinical depression and subclinical depression is approximately twice as high in people with diabetes as in the general population [9,10]. Diabetic patients had a 31% increase in depressive symptoms, compared to 14% of non-diabetic patients [9]. Moreover, because the long–term prognosis of diabetes is highly dependent on appropriate patient self-care behaviors, the clinical correlation between depression and the course of diabetes is also very close. Despite the fact that there has been much research on depression, the neurobiology of depression is still poorly understood due to the lack of specific biomarkers and uncertainty as to whether external stimuli cause depression [11,12]. Additionally, antidepressant efficacy has long been a source of controversy and is part of the reason depression is so difficult to treat. For instance, some medications boost monoamine levels but have no antidepressant effects; not all depressed patients respond to the same antidepressants; antidepressants boost monoamine levels in depressed patients’ brains in just a few hours, but it takes 2–6 weeks for their antidepressant effects to manifest, etc. Therefore, the prime objective of current research on depression is to understand its pathophysiology.

Recently, in reports on depression, there is consensus that depression is associated with neuronal atrophy, and activation of neurons was found to suppress depression and anxiety-like behaviors [13]. Furthermore, depression has been linked to a decrease in astrocytes and the indicators with which they are related. In reaction to synaptically-generated neurotransmitters, astrocytes can establish bidirectional communication with neurons, which in turn releases gliotransmitters that have an impact on synaptic and neuronal activity. By controlling glucose metabolism, neurotransmitter absorption, synaptic formation and maturation, and the blood-brain barrier (BBB), astrocytes also play a significant role in the environment that neurons inhabit [14]. Gene expression in astrocytes can be influenced by neural activity and neuronal activity, which in turn can impact astrocyte development and metabolism. For these reasons, we can infer that dysfunction of astrocytes and neurons, and the abnormal interaction between them, may be the physiopathological basis of depression.

## 2. Neurons and Depression

The monoamine neurotransmitter serotonin hypothesis is the most researched of the current pathogenesis of depression theories. It was shown that 5-hydroxytryptamine (5-HT) modulates the development and excitability of normal neurons [15,16]. This finding raised the possibility that a neuronal basis for depression may also exist. In agreement with this, numerous recent research studies into depression also mention alterations to neuronal function.

**Figure 1:**
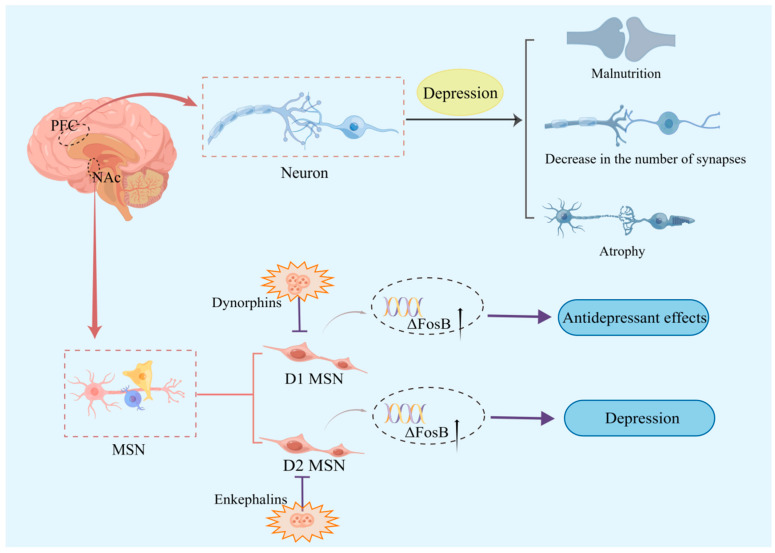
Association of neurons with depression. D1 MSN activity may enhance antidepressant benefits, but D2 MSN activation may exacerbate depressed symptoms. Dynophins block D1-MSN antidepressant effects, while enkephalins block D2-MSN enhancement of activity. Additionally, higher ΔFosB expression was discovered in the D1 MSN of more adaptable mice and in the D2 MSN of less adaptable animals. Furthermore, animal models of depression showed signs of neuronal malnutrition, atrophy, and a reduced number of connections.

One of the two typical features of depression is a pleasure deficit, which is associated with reward system malfunction. The main projection neurons of the nucleus accumbens (NAc) are the middle spiny neurons (MSN), which play a significant role in regulating mood, motivation, and reward circuits. Interestingly, research suggests that activation of the dopamine receptor 1 (D1) MSN may promote antidepressant effects, while activation of the dopamine receptor 2 (D2) MSN exacerbates depressive symptoms [17]. One possible explanation is that this is due to differences in excitatory transmissibility in the NAc MSN subtypes. Dynorphins and enkephalins are neuropeptides present in both neurons, with dynorphins mainly present in D1 MSN, and enkephalins mainly present in D2 MSN [18]. Dynorphin levels in NAc increased [19], while enkephalin levels decreased [20] in a depressed animal model. Enkephalins may help depressed patients return to normal because their level is raised after antidepressants have been taken [21]. Furthermore, preventing the activation of dynorphins may have an antidepressant effect [22]. This alteration may have occurred because enkephalins hinder the augmentation of D2 MSN activity, while dynorphins prohibit D1 MSN from acting as an antidepressant. Moreover, it was demonstrated that the expression of the ΔFosB gene in the NAc of depressed patients is markedly diminished [23], and that it may mediate mice D1 MSN or D2 MSN-mediated adaptation to external stimuli (Figure 1). The D1 MSN of the more adaptable mice [24] and the D2 MSN of the less adaptable mice both showed increased ΔFosB expression. Second, overexpression of ΔFosB in D1 MSN enhanced AMPA receptor (AMPAR) GluR2 expression, which led to a quicker recovery from chronic social defeated stress (CSDS) depression in mice models [23].

Additionally, animal chronic unpredictable mild stress (CUS) models revealed reduced numbers and functional impairments of spiny synapses in pyramidal neurons in the medial prefrontal cortex (PFC) of the brain [25]. CUS also causes neuronal dystrophy [26,27], while binding stress causes atrophy and decreased density of PFC neurons [26,27,28,29,30]. A decrease in the number of GABAergic neurons in the dorsolateral (dl) PFC [31,32], atrophy of pyramidal neurons [31], and changed cell body size of hippocampal neurons [33] were also discovered in postmortem examinations of patients with major depressive disorder (MDD) (Figure 1). This implies that changes in GABA and glutamate circulation levels found in MDD patients may be caused by changes in neurons releasing these neurotransmitters. Therefore, changes in these neurons may lead to modifications in the neurotransmitters they release, resulting in excitatory or inhibitory effects on mood, which may contribute to the pathophysiology of depression.

## 3. Astrocyte and Depression

The central nervous system (CNS) has three different types of glial cells, with astrocytes being the most prevalent and adaptable form; these are further subdivided into protoplasmic and fibrous types. Astrocyte terminals work together with vascular endothelial cells to preserve BBB integrity and give the brain a homeostatic environment [34]. Glial dysfunction in MDD pathogenesis was demonstrated using investigations in animals, postmortem brain examinations, and imaging studies in depressed individuals [35,36,37]. In addition, astrocytes seem to be involved in the process of physical exercise to improve depression. Physical activity has been reported to have antidepressant effects [38]. Studies have shown that people with high levels of physical activity are 17% less likely to develop depression than those with low levels of physical activity [38], and that low physical activity is associated with an increased risk of depression [39]. Physical activity can also reduce the symptoms of depression and serve as a useful supplement to medication and psychotherapy for depression [40,41,42,43]. Physical exercise promotes morphological and functional changes in the brain, acting not only on neurons but also on astrocytes [44]. The effects of physical exercise on astrocytes include an increase in the number of new astrocytes, maintenance of basal levels of catecholamine, increased glutamate uptake, release of trophic factors, and better coverage of astrocytes in the cerebral vasculature [44]. However, little is known about the molecular processes by which astrocytes control depressed behavior.

### 3.1. Astrocyte-Mediated Neuroinflammation in Depression

**Figure 2:**
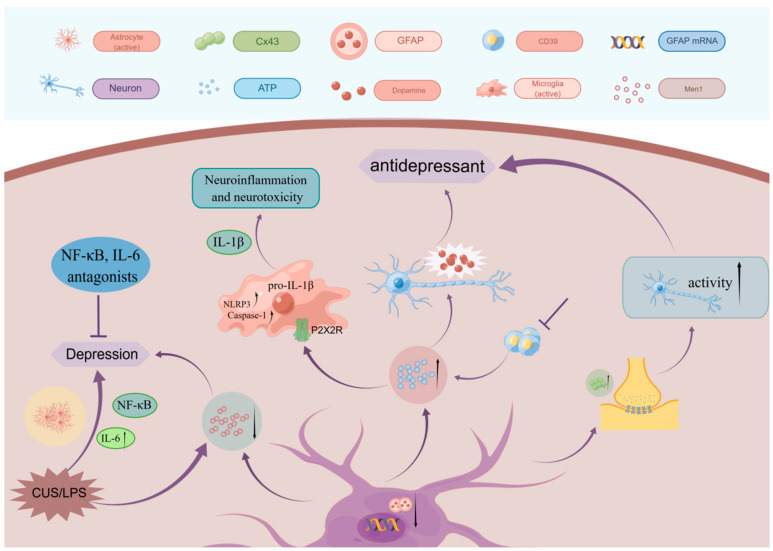
Association of astrocytes with depression. Reducing astrocyte-specific Men1 and lps-induced astrocyte activation both cause depression-like behavior, which can be ameliorated by inhibiting astrocyte activation. Additionally, GFAP, ATP, and connexin alterations were discovered with the onset and treatment of depression.

The neuroinflammatory response is a progressive and complex process, mainly manifested by the activation and proliferation of glial cells, the infiltration of peripheral inflammatory cells and the expression of related inflammatory cytokines. An increasing number of findings support the occurrence of typical neuroinflammatory alterations in depression, primarily in the form of microglia activation, which may also be accompanied by astrocyte activation and altered chemokine levels [45,46]. On the one hand, astrocytes exhibit anti-inflammatory effects by mediating signaling pathways like TGF-β, IFN-γ, and BDNF [47], while on the other, they have pro-inflammatory effects by mediating signaling pathways like TrκB, NF-κB, and VEGF. Leng et al. [48] demonstrated that the expression of multiple endocrine tumor type 1 (Men1) proteins was reduced in the brains of animals exposed to CUS or lipopolysaccharide (LPS), and that the decrease in astrocyte-specific Men1 proteins led to depression-like behavior. The study also discovered decreased Men1 and increased NF-κB activation and interleukin-1β (IL-1β) production in astrocytes. It further discovered depression-like behavior in mice could be improved by NF-kB inhibitors or IL-1b receptor antagonists. The idea that astrocyte-mediated neuroinflammation is related to the pathology of depression is supported by several investigations. LPS-induced astrocyte activation can lead to depressive-like behaviors that can be alleviated by suppressing astrocyte responses [49,50,51] (Figure 2).

In conclusion, astrocyte-mediated neuroinflammation is critical for the onset and development of depression. Few clinical studies have been conducted on the neuroinflammatory aspects of depression, and most of those studies concentrate on microglia activation rather than astrocyte activation or other aspects. It is, therefore, necessary to focus more attention in the future on the activation of astrocytes to find more therapeutic targets for depression.

### 3.2. Astrocyte-Related Markers in Depression

#### 3.2.1. Adenosine Triphosphate (ATP)

The most direct source of energy in living organisms is ATP, which can be released by neurons and astrocytes, and maintained in certain concentrations outside the cell. Vesicular ATP, produced by astrocytes, is a significant source of extracellular ATP. Deficient ATP release from astrocytes was observed in animal models of depression [52,53] and is also related to abnormal synaptic plasticity in depression [52,54,55,56,57].

Upon release into the extracellular compartment, ATP concentration is regulated by the ectonucleotide tris(di)phosphate hydrolase (ENTPD). The ENTPD family consists of seven different isoforms (ENTPD1-6 and ENTPD8), and CD39 belongs to an ENTPD1 isoform [58]. It was found that the chronic social defeated stress (CSDS) model enhanced hippocampal CD39 expression and activity, and that pharmacological inhibition and gene silencing of CD39 could exert antidepressant effects by raising hippocampal extracellular ATP concentrations [59]. Additionally, Cao et al. discovered that CSDS model mouse prefrontal cortex ATP levels were significantly decreased, and ATP injection into the medial PFC (mPFC) produced an antidepressant effect. According to this study, ATP must bind to the P2 × 2 receptor (P2 × 2R) in the mPFC in order to have an antidepressant effect [52]. ATP released from astrocytes also modulates the release of neuronal dopamine (DA), and the reward circuit mediated by dopamine is closely associated with depression [60] (Figure 2). As a result, astrocyte-produced ATP has the potential to function as an antidepressant.

#### 3.2.2. Glial Fibrillary Acidic Protein (GFAP)

Eng initially extracted and described GFAP from mature astrocytes in 1969 [61]. As a member of the cytoskeletal protein family and a significant part of the cytoskeletal intermediate filament, GFAP controls the movement and shape of astrocytes by offering structural stability during their development [61].

It was demonstrated that GFAP-immunoreactivity (GFAP-IR) in depressed patients younger than 60 years showed significantly lower GFAP area fractions in their prefrontal cortex, dorsolateral gray matter, and orbitofrontal cortical gray matter [62,63]; in contrast, increased GFAP-IR area fraction and cell density were found in the dorsolateral prefrontal cortical gray matter of older depressed patients [62,64]. This shows that astrocyte expression differs between young and older depressed individuals in the cortical gray matter, and that this difference may be the result of an adaptive response to neuronal damage in older depressed patients [65]. Additionally, patients with MDD were reported to have decreased mRNA [66,67], protein levels [63,67,68], and GFAP isoforms [69] (Figure 2). In conclusion, the above changes in astrocytes associated with depression indicate abnormal astrocyte function in depressed patients.

Treatment for depression has also been linked to changes in astrocytes. One study found that fluoxetine prevented psychosocial stress-induced reductions in the number of astrocytes [70], while another study found that riluzole can prevent the reduction in GFAP mRNA expression in the rat prefrontal cortex after exposure to CUS [71]. Citalopram and fluoxetine, two SSRI antidepressants, are thought to possess antidepressant effects because they can stimulate calcium signaling in astrocytes in the prefrontal cortex [72].

In conclusion, the astrocyte marker GFAP and its mRNA were changed in both depressed patients and animal models, and downregulation of GFAP mRNA in animal models could be reversed by electroconvulsive treatment. Therefore, GFAP could be the focus of subsequent studies on the association between depression and astrocytes and become a new potential target for antidepressant drugs to act.

#### 3.2.3. Connexins

According to recent studies, the improvement to the gap junction dysfunction in astrocytes may be associated with depression therapy [73]. Connexins make up the gap junction channels (GJCs), of which connexin 43 (Cx43) is the most prevalent. The gap junctions between astrocytes are essential for information exchange: when the level of connexin decreases, the gap junctions stop functioning properly, inhibiting normal intercellular communication, and leading to abnormal brain circuits.

Mitterauer et al. made the initial claim that decreased connexins are connected with the pathophysiology of depression [74]. In earlier studies, the locus coeruleus (LC), prefrontal cortex (PFC), and hypothalamus were reported to have lower levels of Cx43 gene expression in MDD patients compared with controls, and the gene expression of Cx43 was also decreased in the orbitofrontal cortex, neocortex, and LC [75]. This indicates that the pathophysiology of depression may be related to connexin expression. In a study on the function of connexins in depression, it was discovered that increasing Cx43 expression in the mPFC of mice exposed to CSDS increased their neuronal activity and alleviated CSDS-induced depression-like behavior. Conversely, inhibiting Cx43 expression in normal mice had the opposite effect [76] (Figure 2). Sucrose preference studies revealed a significant decrease in the amount of sucrose ingested after injection of the non-selective GJC inhibitor carbenoxolone (CBX) into the PFC of normal rats, indicating reduced pleasure in rodents [77]. In addition, long-term fluoxetine or duloxetine treatment in rats reversed the stress-related decrease in Cx43 levels [78].

In summary, alterations in connexins have been found in both patients with depression and animal models of depression, and the results of animal studies support a role for connexins in the pathogenesis and treatment of depression. Connexins are mainly expressed in glial cells, especially astrocytes; therefore, astrocytes are key intermediates if we wish to further investigate the relationship between connexins and depression.

## 4. Astrocyte–Neuron Interactions

The “tripartite synapse” is a concept used to describe the interaction between astrocytes and neurons; the concept proposes that the synapse is made up of three parts, including the pre- and postsynaptic nerve ends, and the terminal protrusions of astrocytes [79]. The tripartite synapse is essential for regulating extracellular fluid, ion homeostasis, ion transport, cerebral blood flow, synaptic remodeling, and energy supply in order to sustain stable neuronal activity [80,81,82,83]. Overall neural homeostasis depends on normal energy metabolism. By releasing glutamate [84], neurons trigger aerobic glycolysis, glycogenolysis, and lactate generation in surrounding astrocytes. Lactate created by astrocytes is then released and transported to neurons by the monocarboxylate transporter protein (MCT) to replenish their energy needs [85,86].

### 4.1. Ca^2+^ Excitability

**Figure 3:**
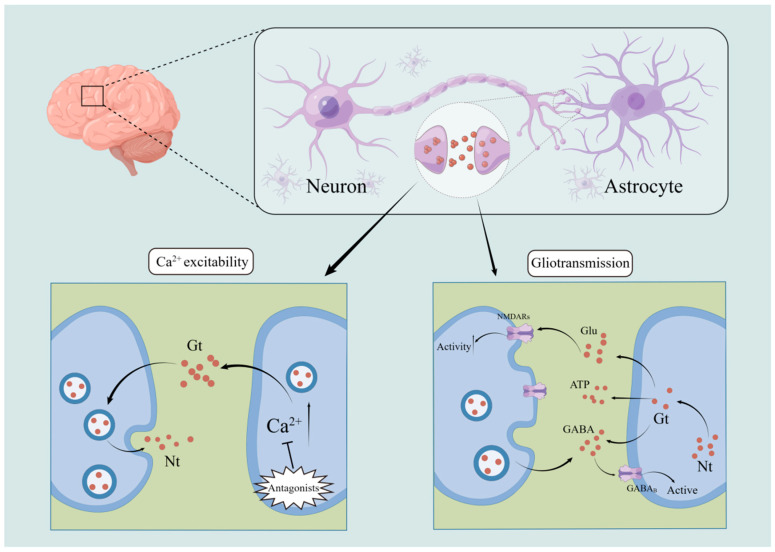
Astrocyte–neuron interactions. Increased Ca^2+^ concentration in astrocytes causes neurons to respond, but this shift can be prevented by the use of ionotropic glutamate receptor antagonists. Depending on the type of receptor it binds, astrocytes can also affect synaptic transmission by releasing various gliotransmitters. At the same time, astrocytes can be activated via GABAB receptors by low-level stimulation of GABAergic interneurons.

It is significant that astrocytes can exhibit excitability in response to variations in intracytoplasmic Ca^2+^ concentration [87,88], because until then astrocytes were thought to be cells that did not generate excitatory impulses. Neurotransmitters released during synaptic activity can stimulate astrocytes, indicating a relationship between neurons and astrocytes [89,90,91,92,93]. Studies have revealed that Ca^2+^ levels in neurons were elevated after the application of external light, electrical, and other stimuli that cause elevated Ca^2+^ levels in astrocytes. This suggests that neurons respond to these elevated Ca^2+^ concentrations in astrocytes [94]. However, when intervening with ionotropic glutamate receptor antagonists, neuronal Ca^2+^ concentrations do not change in response to increased astrocyte Ca^2+^ concentrations (Figure 3). This also reflects the dependence on glutamate for information exchange between astrocytes and neurons [94]. Moreover, astrocytes may exhibit Ca^2+^ variations in excitability in response to external sensory stimuli. It was demonstrated that touching a mouse’s whiskers causes it to produce more Ca^2+^ [90]; that stimulating a mouse’s eyes causes astrocytes in the visual cortex to produce more Ca^2+^ [93]; and that electrically stimulating the nucleus accumbens causes astrocytes in the sensory cortex to produce more Ca^2+^ in response to sudden external stimuli [91]. In summary, astrocytes in the brain of the respective sensory sites react to the stimuli by raising Ca^2+^ concentrations when external sensory stimuli are applied to various places. As a result, it is possible that astrocytes and neurons are involved in the processes by which the brain responds to the external environment.

### 4.2. Gliotransmission

During signaling between neurons and astrocytes, astrocytes actively regulate synaptic and neuronal activity, as well as respond to neuronal activity. It was previously believed that astrocytes might passively maintain the balance of synaptic transmission through mechanisms including potassium buffering and transmitter scavenging. Recent research, however, has revealed that astrocytes also have significant effects on the neuronal microenvironment through mechanisms like glucose metabolism control, neurotransmitter absorption, synaptic growth and maturation, and the BBB. Notably, gliotransmission—the process by which astrocytes alter neuronal and synaptic function by releasing neuroactive chemicals like glutamate, ATP, and GABA [95]—was discovered to be the mechanism by which astrocytes act.

Many hypotheses have been developed about how astrocytes release gliotransmitters, among which there is more agreement on a calcium- and trap protein-dependent mechanism [96,97,98,99] whereby astrocytes depend on calcium and trap proteins to regulate the release of gliotransmitters from vesicles. Neurotransmitters can stimulate astrocytes to release gliotransmitters, but not all of them have this effect. Both purinergic receptor P2Y1 and protease-activated receptor 1 (PAR1), for instance, cause an increase in calcium levels in the astrocytes of the hippocampal nucleus [100], but only PAR1 receptors cause astrocytes to release gliotransmitters, because glutamate released by astrocytes during this process activates N-methyl-D-aspartate receptor (NMDAR) neurons [101]. Depending on the type of receptor it contacts, the various gliotransmitters generated by astrocytes can potentially affect synaptic communication. In the hippocampus, for instance, glutamate produced by astrocytes can act on postsynaptic NMDAR to increase neuronal excitability; however, it can also bind to presynaptic class II–III metabotropic glutamate receptors to cause heterosynaptic inhibition [102]. This shows that astrocytes act by releasing gliotransmitters that activate different neuronal receptors. In addition, on the one hand, astrocytes are stimulated to create glutamate as a result of the release of endocannabinoids from neurons, which allows them to communicate with neurons located further away [103]. On the other hand, ATP produced by astrocytes not only works as a bridge for communication between astrocytes and neurons, it also acts as a bridge for neurons to transmit information extrasynaptically [104] (Figure 3). This implies that information may also be transmitted to far-off synapses through interactions between astrocytes and neurons.

Astrocytes can release different gliotransmitters to affect neurons, while multiple neuronal stimuli can also affect astrocytes [105]. It has been demonstrated that GABAergic interneurons can activate astrocytes via GABA_B_ receptors when they are stimulated at low levels, but high levels of stimulation result in a biphasic response (Figure 3). This implies that astrocytes are also capable of responding to information received from neurons, and transmitting it via certain gliotransmitters, thus exerting an excitatory or inhibitory effect on synaptic transmission.

Gliotransmission is also associated with the proper functioning of the microbiota–gut–brain axis. According to the gut microbiota hypothesis, the gut microbiota can influence neurotransmitter synthesis and recognition [106,107,108], and affect the brain and behavior through the gut–brain axis. The brain–gut axis is a bidirectional information conversion pathway between the mammalian brain and intestine, which connects the brain and the intestine through multiple pathways including the neural, hypothalamus–pituitary–adrenal (HPA) axis, and the immune system [109,110]. Factors such as psychological stress and illness can damage one or more pathways of the brain–gut axis, which can cause brain–gut axis dysfunction and lead to depression [111,112]. It was found that the gut microbiota of depressed patients was significantly different from that of healthy controls, i.e., the diversity and richness of the microbiota in depressed patients was reduced [113,114]. In addition, gut microbiota disorders increase susceptibility to depression, while restoration of gut microbiota can alleviate depression; depressive symptoms can also be transmitted after fecal microbial transplantation [115].

In the final analysis, gliotransmitter transmission between astrocytes and neurons is a complicated phenomenon. Despite extensive research on gliotransmitters, the diversity of signals received and sent between them remains unknown, as does whether various gliotransmitters are released simultaneously or by the same astrocyte, and how they affect the microbiota–gut–brain axis. Therefore, it is essential to understand the mechanisms underlying the release of various gliotransmitters, and the circuits through which they communicate with astrocytes and neurons.

### 4.3. Astrocyte–Neuron Interactions in Depression

Because it is crucial for mood regulation, the interaction between astrocytes and neurons also affects the onset and progression of depression. It was proposed that gliotransmitters generated by astrocytes may regulate excitatory and inhibitory neurotransmission in the central amygdala (CeM) [116]. The basolateral amygdala (BLA) and CeM, which make up the majority of the amygdala, are crucial for the control of fear and anxiety. According to research by Martin Fernandez et al. [116], selective activation of astrocytes in the medial subregion of the CeM allows for the activation of A1 adenosine receptors, which have an inhibitory effect on excitatory synapses in the BLA; and A_2A_ receptors, which have an enhancing effect on inhibitory synapses in the lateral branches of the CeM. Decreasing the firing rate of CeM neurons also reduced the expression of worry and terror. Astrocyte–neuron signaling is, therefore, a synaptic transmission and circuit conduction process. This phenomenon has important physiological implications, which imply that cooperation between astrocytes and neurons modulates anxiety and fear responses.

Further, the interaction between astrocytes and neurons may be related to the burst of neuronal activity in the lateral habenula (LHB) in depression. LHB is the brain’s “anti-reward center,” which is crucial for encoding negative rewards [117,118,119]. According to studies [120,121], animals experience depressive symptoms after repeated CUS, while at the same time, LHB neurons become abnormally active. However, depressed symptoms can be successfully reversed by lowering, or rather attenuating, this aberrant activity of LHB neurons. Cui et al. [120] demonstrated that astrocyte–neuron interactions may be responsible for the depression-related immediate increase in LHB neuronal activity. This study discovered a strong correlation between the level of the potassium channel Kir4.1 on astrocytes and the degree of membrane hyperpolarization and activity of LHB neurons. When the potassium channel Kir4.1 in the astrocyte membrane surrounding the LHB neuron was upregulated, the LHB neurons displayed a sudden increase in activity and the rats showed depressive symptoms; whereas the opposite occurred when Kir4.1 was downregulated. This shows that Kir4.1 on astrocyte membranes may be a new target for the treatment of depression and that astrocyte–neuron interactions in the LHB may be responsible for the spike in neuronal activity seen in depression. As a result, it is likely that astrocyte–neuron interactions have a role in depression. A number of recent studies have also shown a link between astrocyte–neuron cooperation and depression; however, the precise mechanism of action is yet unknown.

#### 4.3.1. Synaptic Plasticity

One of the most fundamental and critical functions in the brain is synaptic plasticity, which describes the experience-dependent changes in synaptic strength. Long-term enhancement (LTP) and long-term depression (LTD) are two major manifestations of sustained changes in synaptic efficacy, and they are thought to be key cellular mechanisms for learning and memory [122]. The regulation of connections between neurons determines whether synaptic transmission is effective or not [123]. Astrocytes are critical for synaptic transmission and plasticity, although it is unclear how their interactions with neurons control synaptic plasticity.

Although previous studies on LTD have concentrated on its connection with neurons, a growing body of current data suggests that astrocytes are also involved in LTD. As components of the tripartite synapse, astrocytes are involved in the brain’s processing of incoming information from the outside world through interactions with neurons and synapses [79]. For example, higher calcium concentrations in astrocytes can trigger the release of gliotransmitters like glutamate, ATP, and d-serine, thereby affecting synaptic transmission [124,125,126]. Increased extracellular concentrations of adenosine [127] generated by astrocytes were found to enhance the suppression of t-LTD in the mouse hippocampus, which led to the disappearance of t-LTD [128,129] and could be plastically converted to t-LTP [128]. It was also demonstrated that calcium signaling by astrocytes and the activation of IP3R2 are necessary for A1R-mediated LTD on mid-spiny neurons at cortical striatal synapses, whose particular Gq–GPCR chemical activation can also induce A1R-mediated LTD [130,131]. At the same time, astrocytes and neurons are strongly associated with LTP, another form of synaptic plasticity expression. The temporal integration of synaptic inputs is controlled by Ca^2+^ communication between neurons and astrocytes. Liu et al. [132] demonstrated that late LTP (L-LTP) but not early LTP (E-LTP) are dependent on astrocyte inositol trisphosphate receptor type 2 (IP3R2)-dependent Ca^2+^ signaling. Thus, LTP and LTD are influenced by both neurons and astrocytes, and both cells play a regulatory function in synaptic plasticity. 

Research by Wang et al. [133] also raises the idea of another mechanism by which astrocytes and neurons could control synaptic plasticity. Their research demonstrated that the activation of the scaffolding protein PSD-95 and the interleukin-33 (IL-33) receptor complex at neuronal synapses can accumulate IL-33, which excites synapses and boosts neurotransmission. In vivo administration of IL-33 promotes the formation of functional excitatory synapses in neurons in the CA1 region of the hippocampus, whereas the specific knockdown of IL-33 in astrocytes in the CA1 region results in reduced excitatory synapses. Inhibiting steady-state synaptic plasticity in CA1 area pyramidal neurons and interfering with the establishment of their spatial memory are both brought about by blocking IL-33 and its receptor signaling in mouse brain. This implies that neurons and astrocytes may mediate homeostatic synaptic plasticity by co-regulating IL-33. 

Research over the past few years has shown that depression is closely related to synaptic plasticity. Long-term reinforcement of synapses in the CA1 region of the hippocampus leads to the emergence of depression-like behaviors, and animal models of depression have also shown reductions in synaptic proteins and growth factors needed for hippocampal LTP. Improvements in synaptic plasticity may also be linked to the antidepressant effects of tricyclic antidepressants (TCA) and selective serotonin-reuptake inhibitors (SSRI) [134]. Stress also reduces LTP in the CA3 and boosts LTD and peak time-dependent LTD (tLTD) in the CA1 [123]. In conclusion, decreased synaptic plasticity is significantly linked to depression, and astrocytes and neurons are crucial for enhancing synaptic plasticity and preserving its homeostasis. Therefore, the synaptic plasticity mediated by astrocytes and neurons should not be neglected in the treatment of depression and its prognosis.

#### 4.3.2. Energy Metabolism

The beginning of depression might be frequently accompanied by changes in the brain metabolic balance, such as abnormalities in neurotransmitter and energy metabolism and changes in glucose metabolism. Antidepressant treatment altered lactate release and glucose consumption in astrocytes in vitro [135,136], raising the possibility that lactate plays a role in depression.

Often in the absence of neural activation, a large rise in lactate in the brain is likely to be a symptom of pathology. To illustrate, increased lactate levels in the blood, brain, and cerebrospinal fluid (CSF) were used as indicators of mitochondrial dysfunction [137,138]. This is because lactate does not accumulate in the brain or CSF when mitochondrial metabolism is intact; whereas, when mitochondrial dysfunction occurs, intracerebral metabolism switches to extramitochondrial glycolysis, and the lactate produced by glycolysis cannot be completely eliminated by mitochondrial metabolism, leading to lactate accumulation [139,140]. It is interesting to note that psychiatric conditions like depression frequently coexist with mitochondrial malfunction. Animal models of depression were also shown to have elevated lactate levels and pathological abnormalities in mitochondrial structure and function [141,142,143].

Astrocyte–neuron interactions play an important role in the energy metabolism of the central nervous system (CNS). On the one hand, the protrusions of astrocytes contain a large number of glucose transporters, which can help them to take up glucose directly from the blood. Astrocytes then produce lactate for neuronal energy through anaerobic enzymes, realizing the astrocyte–neuron lactate shuttle (ANLS) [144]. However, astrocyte energy deficiency leads to reduced dendritic branching, increased neuronal sensitivity, and increased susceptibility to depression [145]. On the other hand, neurons in ANLS are able to influence gene transcription in astrocytes, thereby inducing lactate export and glucose metabolism, and controlling the homeostatic regulation of astrocyte metabolic fluxes [146]. Vasoactive intestinal peptides secreted by neurons bind to vasoactive intestinal peptide receptors on the surface of astrocytes, boosting astrocyte glycogenolysis [144]. Additionally, neurons increase astrocyte lactate production through the cAMP/PKA pathway [146]. Yin et al. [147] revealed that during forced swimming trials, the extracellular lactate in the mouse brain momentarily increases as a consequence of being newly created in astrocytes. Moreover, Carrard et al. [148] and Karnib et al. [149] discovered that peripheral lactate delivery induced antidepressant-like effects. Lactate administration can increase the concentration of 5-HT by increasing the 5-HT receptor binding protein p11; it can also increase the expression of the astrocyte marker S100β. This suggests that astrocytes may modify the antidepressant-like effects of lactate by controlling the transport of 5-HT [141,148]. Furthermore, paroxetine and fluoxetine, two common antidepressants, can increase glucose metabolism and reduce astrocyte glycogen production, which helps the nervous system recover and alleviate depression symptoms [150]. As a consequence, one of the key elements influencing the onset of depression may be the disturbance of energy metabolism in the brain caused by astrocyte–neuron dysfunction.

## 5. Conclusions and Prospects

Although we have recently moved into a new phase of understanding in depression research, its etiology is still unknown. Because of variances in the people being stimulated, there are variations in how external stimuli are processed in the brain before they are turned into a depressed state, as well as in the reasons why external stimuli do not transform into a depressed state in some people. Depression has been linked to changes in neurons and astrocytes, as previously indicated (Table 1). First, depressed people exhibit neuronal loss, atrophy, and lower density, and MSN may be correlated with the severity of depression. Second, astrocyte-mediated neuroinflammation may be involved in the pathogenesis of depression, and research into depression has also revealed alterations in astrocyte-associated substances, typically ATP, GFAP, connexins, etc. Finally, communication between neurons and astrocytes can occur via Ca^2+^ conductance and gliotransmission. This connection between neurons and astrocytes regulates anxiety and fear responses, and may also be responsible for the spike in neuronal activity that occurs during depression. Additionally, they affect alterations in synaptic plasticity and energy metabolism that are connected with depression. It follows that it is highly possible that the interaction between neurons and astrocytes is very important in the initiation and progression of depression. 

Based on the above, we propose the theory that when an individual is subjected to external stimuli, Ca^2+^ in the astrocytes of the sensory cortex is altered and further influences neurons, contributing to the pathophysiological process of depression. Alternately, neurons and astrocytes may contribute to depression by co-regulating the levels of specific neurotransmitters through gliotransmission and influencing synaptic plasticity in addition to the energy metabolism associated with depression. In vivo cell-to-cell conversion has also lately become a novel disease-treating strategy. Depression is characterized by a loss of neurons, and a study by Qian et al. [151] revealed that removing the RNA-binding protein PTB (PTBP1) from astrocytes can transform astrocytes into functioning neurons, suggesting a potential new depression treatment. All things considered, better understanding of the pathophysiological mechanisms underlying astrocyte–neuron interactions in depression may hold the key to its prevention, treatment, and the creation of new drugs.

## Figures and Tables

**Table 1:** Marker substances in depression linked to astrocytes and neurons. NAc: Nucleus accumbens; D1: Dopamine receptor 1; D2: Dopamine receptor 2; Men1: Multiple endocrine tumor type 1; IL-1β: Interleukin-1β; ATP: Adenosine triphosphate; PFC: Prefrontal cortex; P2 × 2R: P2 × 2 receptor; GFAP: Glial fibrillary acidic protein; LC: Locus coeruleus; LHB: Lateral habenula; 5-HT: 5-hydroxytryptamine. ↑: Expression upward. ↓: Expression downward.

Location	Markers	Level	Associated Receptors	Be Related with
Neuron	Dynorphins	NAc ↑ [19]	D1	Lack of pleasure [17]
	Enkephalins	NAc ↓ [20]	D2	
	ΔFosB	NAc ↓ [23]	/	Adaptability [24]
Astrocyte	Men1	Brain ↓ [48]	/	Neuroinflammation [48]
	IL-1β	↑ [48]	IL-1β receptors	
	ATP	PFC ↓ [52]	P2 × 2R	Pleasure [60]
	GFAP	PFC ↓ (Under 60 years of age) [62,63]	/	Occurrence of depression [62,63,64]
		PFC ↑ (Over 60 years of age) [62,64]	/	
	Connexins	LC, PFC and hypothalamus ↓ [75]	/	GJCs [77]
Neurons and Astrocytes	Kir4.1	LHB ↑ [120]	/	Negative rewards [117,118,119]
	Lactate	Brain ↑ [147]	5-HT receptors	Energy metabolism [146]

## Data Availability

No data were used for this review.
